# Supplementary material for: Preparation and Characterization of Pickering Emulsions with Modified Okara Insoluble Dietary Fiber
Source: Foods. 2021 Dec 3;10(12):2982. doi: 10.3390/foods10122982 (PMC8700857; doi:10.3390/foods10122982)
Supplement: Supplementary file 1 [file foods-10-02982-s001.zip › foods-1448123-supplementary.pdf]

Supplementary material

Table S1. Optimization of OIDF-Pickering emulsion preparation treatment parameters

Treatement	Ultrasonic power (W)	Ultrasonic time (min)
1	300	3
2	400	3
3	500	3
4	600	3
5	700	3
6	500	3
7	500	6
8	500	9
9	500	12
10	500	15

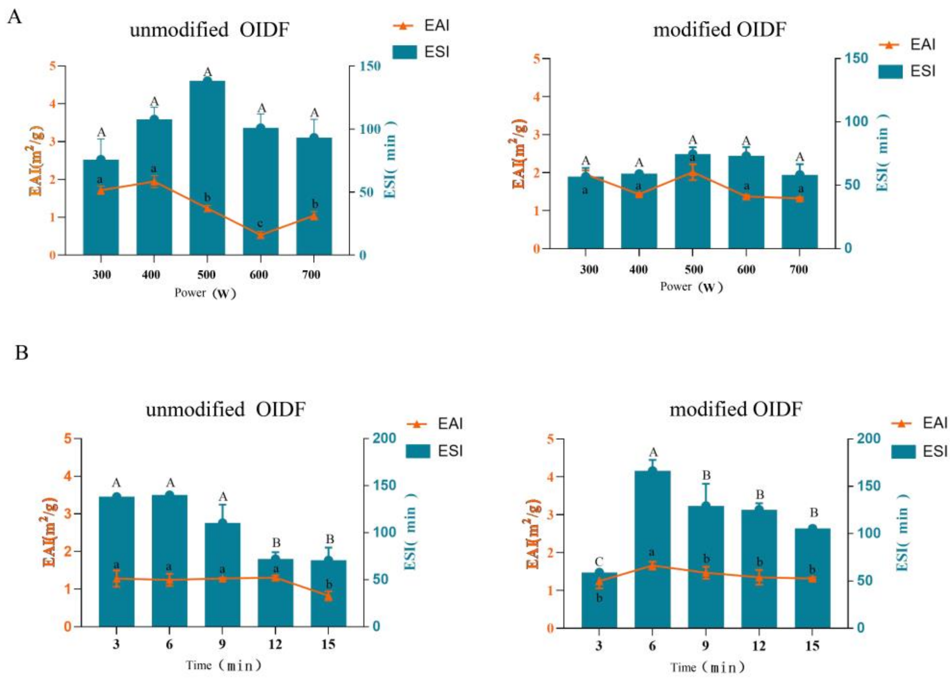

Figure S1 Emulsifying ActivityIndex (EAI) and Emulsifying Stability Index (ESI) of OIDF-Pickering emulsions. (A) EAI and ESI of OIDF-Pickering emulsions at different ultrasonic power; (B) EAI and ESI of OIDF-Pickering emulsions at different ultrasonic time.
